# Forschungsprojekt EUBeKo: Entscheidungs- und Umsetzungsprozesse verhältnisorientierter Bewegungsförderung in der Kommune für mehr Chancengerechtigkeit systematisch planen und implementieren

**DOI:** 10.1007/s11553-023-01036-5

**Published:** 2023-04-29

**Authors:** Lisa Paulsen, Lea Benz, Izabela Bojkowska, Bruno Domokos, Christina Müller, Birgit Wallmann-Sperlich, Jens Bucksch

**Affiliations:** 1 Abteilung Prävention und Gesundheitsförderung, Fakultät für Natur- und Gesellschaftswissenschaften, Pädagogische Hochschule Heidelberg, Heidelberg, Deutschland; 2 Institut für Sportwissenschaft, Julius-Maximilians-Universität Würzburg, Würzburg, Deutschland

**Keywords:** Stadt, Land, Gesundheitsförderung, Intervention, „Change agents“, Multiplikator:innen, Urban, Rural, Health promotion, Intervention, Change agents, Multipliers

## Abstract

**Hintergrund:**

Bewegung ist über eine Reihe sozialökologischer Bedingungsfaktoren zu verstehen, an welchen eine erfolgreiche Bewegungsförderung ansetzen muss. Kommunen nehmen dabei eine bedeutende Rolle ein, da sie gesundheitsförderliche Verhältnisse ermöglichen können. Häufig wird die Konzipierung, Umsetzung und Evaluierung sozialökologischer Ansätze jedoch nicht systematisch und theoriegeleitet abgeleitet. Veränderungen in den Verhältnissen werden durch das Verhalten und die Entscheidungen sog. „change agents“ bzw. kommunaler Entscheidungstragender, wie z. B. Bürgermeister:innen, beeinflusst. Daher ist es wichtig, Einflussfaktoren auf Entscheidungsprozesse zu kennen, um Überzeugungsarbeit für Bewegungsförderung in der Kommune zu leisten. Zudem braucht es Multiplikator:innen (z. B. Mitarbeitende in Gesundheitsämtern), die Kompetenzen besitzen, verhältnisorientierte Interventionen in Kommunen systematisch umzusetzen.

**Zielstellung und Projektverlauf:**

Das Forschungsprojekt „Entscheidungs- und Umsetzungsprozesse verhältnisorientierter Bewegungsförderung in der Kommune für mehr Chancengerechtigkeit systematisch planen und implementieren“ (EUBeKo) wurde im Rahmen des Förderschwerpunkts „Bewegung und Bewegungsförderung“ des Bundesministeriums für Gesundheit gefördert. In diesem Beitrag werden das Projekt EUBeKo mit seinen zwei Forschungsfragen zum einen nach der Rolle und den Kompetenzen von Multiplikator:innen und zum anderen nach den Einflussfaktoren auf kommunale Entscheidungsprozesse sowie die Planung und Umsetzung verhältnisorientierter Bewegungsförderung in zwei Modellkommunen als auch die Strategien der Dissemination (z. B. Projekt-Webseite) beschrieben.

**Diskussion und Schlussfolgerungen:**

Zu den Stärken des Projekts zählt die systematische und theoriegeleitete Aufbereitung und Durchführung eines Prozesses verhältnisorientierter Bewegungsförderung in der Kommune mit besonderem Fokus auf Planungs- und Entscheidungsprozesse sowie auf die Zielgruppen der kommunalen Multiplikator:innen und Entscheidungstragenden. Herausforderungen finden sich im Theorie-Praxis-Transfer und in den Auswirkungen der COVID-19-Pandemie („coronavirus disease 2019“) auf die Projektumsetzung.

## Abstract (en)

**Background:**

Physical activity can be understood in terms of socioecological determinants, which are prerequisites to successfully promote physical activity. Communities play an important role in this context, as they can create health-protective conditions. However, the design, implementation, and evaluation of socioecological approaches often do not follow a systematic and theory-driven approach. Changes in environmental conditions are determined by the behavior and decisions of so-called change agents or local decision-makers, such as mayors. Therefore, it is important to identify the determinants that influence decision-making processes to be able to change the decisional behavior of change agents to promote physical activity in their communities. In addition, there is a need for multipliers (e.g., employees in health offices) to be qualified to systematically implement socioecological-based interventions in communities.

**Objectives and project process:**

The research project “Systematic planning and implementation of decision-making and implementation processes of socioecological-based physical activity promotion in the community to reduce social inequity” (with the German acronym EUBeKo) was funded within the framework of the funding scheme “Physical Activity and Physical Activity Promotion” of the German Federal Ministry of Health. It takes both the local change agents and multipliers into account and is inspired by intervention mapping. This article describes the project EUBeKo with its two research questions on the role and competences of multipliers, and on the factors influencing local decision-making processes as well as the planning and implementation of socioecological-based physical activity promotion in two model communities, and the dissemination strategies (e.g., project website).

**Discussion and conclusions:**

The strengths of the project include the systematic and theory-based preparation and implementation of a process of socioecological-based physical activity promotion in the community with a special focus on planning and decision-making processes as well as on the target groups of local multipliers and decision-makers. Challenges are found in the theory–practice transfer and the impact of the coronavirus disease 2019 (COVID-19) pandemic on project implementation.

## Hintergrund

Regelmäßige Bewegung ist eine Voraussetzung für die Förderung und den Erhalt von Gesundheit. Allerdings bewegt sich die deutsche Bevölkerung zu wenig und sitzt zu lange [11]. So zeigen aktuelle Daten, dass weniger als die Hälfte der Männer und Frauen (48,0 % bzw. 42,6 %) die ausdaueraktivitätsorientierte Bewegungsempfehlung der Weltgesundheitsorganisation (WHO) von mindestens 150 min pro Woche (zur genauen Definition s. [40]) erreicht. Werden zusätzlich noch die muskelkräftigende Bewegung hinzugenommen, sind es nur ca. ein Fünftel der Frauen (20,5 %) und ein Viertel der Männer (24,7 %), die die Empfehlung von zwei Krafttrainings pro Woche vollständig erreichen [11].

Bewegungsförderung ist insgesamt erfolgreicher, wenn sie theoriebasiert an den sozialökologischen Bedingungsfaktoren des Bewegungsverhaltens ansetzt [8, 14, 35]. Diese Faktoren liegen beispielweise auf individueller (z. B. Werte und Einstellungen) und umweltbezogener Ebene [13]. Die Umweltebene wird zumeist aufgeteilt in weitere Ebenen, wie z. B. eine soziokulturelle (z. B. soziale Unterstützung), eine physische (d. h. eine baulich-technische und natürliche [z. B. Flächennutzung der Wohnumgebung]) sowie eine politische und ökonomische (z. B. Gesetzgebung und Fördermittel; [3, 8, 39]). Häufig werden individiumsbezogene Ansätze in Prävention und Gesundheitsförderung (sog. Verhaltensprävention) priorisiert, wie z. B. Programme zur Motivationssteigerung oder Einstellungsveränderung, obgleich nur ein eingeschränkter Erfolg (z. B. geringe Effektgrößen und Teilnahmequoten sowie mangelnde Nachhaltigkeit) nachgewiesen werden kann [30]. Gerade vulnerable Gruppen, die besonders von Maßnahmen der Prävention und Gesundheitsförderung profitieren würden, fühlen sich durch verhaltensbezogene Angebote häufig nicht angesprochen oder der niederschwellige Zugang zu den Angeboten fehlt. Eine gesundheitliche Chancengerechtigkeit ist damit schwer erreichbar [19]. In den Fokus rücken deshalb verstärkt Ansätze der Verhältnisprävention auf der Umweltebene, die die Lebensumstände der Bevölkerung nachhaltig beeinflussen [4, 16, 27].

Kommunen bzw. Quartiere können zentrale Lebenswelten für verhältnispräventive Interventionen der Gesundheits- und Bewegungsförderung darstellen. In Kommunen können über die Verhältnisprävention alle Zielgruppen, insbesondere auch vulnerable Zielgruppen wie Arbeits- oder Wohnungslose, bildungsferne Gruppen oder Menschen mit Behinderung, erreicht werden. Auch die Nationalen Empfehlungen für Bewegung und Bewegungsförderung betonen das Erfolgspotenzial politik- und verhältnisorientierter Interventionen [32]. Eine abschließende Evidenzbewertung für kommunale Interventionen, die mehr als nur eine Ebene sozialökologischer Modelle einbeziehen, liegt jedoch u. a. aufgrund von Herausforderungen in der Durchführung von adäquaten Studien noch nicht vor [1, 2, 12]. Die Nationalen Empfehlungen für Bewegung und Bewegungsförderung sehen auf Bevölkerungsebene für gemeindebezogene, Mehrkomponenten-, umweltbezogene und politikbezogene Ansätze eine eingeschränkte mittlere Evidenzstufe [32]. Ein möglicher Grund für Schwierigkeiten in der Konzipierung, Umsetzung und Evaluierung sozialökologischer Ansätze liegt häufig in einer fehlenden Beachtung systematischer und theoriegeleiteter Interventionsplanung, sodass Wirkmechanismen der Interventionen verdeckt bleiben sowie die Replizierbarkeit und Beurteilung der Wirksamkeit nicht in angemessener Weise möglich sind [3, 24, 29].

Das „intervention mapping“ ist ein Planungsmodell mit sechs Kernschritten, das eine sozialökologische Fundierung hat, explizit verhältnisbezogene Determinanten berücksichtigt und dabei hilft, diese in Interventionsmethoden und kreative Maßnahmen zu übersetzen [3, 20, 28]. Laut „intervention mapping“ werden Umweltbedingungen durch das Verhalten und die Entscheidungen sog. „change agents“ (CA) beeinflusst. CA agieren auf verschiedenen Umweltebenen des sozialökologischen Modells und sind (meist) nicht persönlich von einem Gesundheitsproblem betroffen, aber ihr Entscheidungsverhalten kann das Verhalten der Zielgruppe beeinflussen [3, 34, 35]. In diesem Beitrag werden CA als Personen mit einer gewissen Entscheidungskompetenz bzw. -befugnis (kommunale Entscheidungstragende) in der Kommunalpolitik und -verwaltung (z. B. Gemeinderatsmitglieder, Bürgermeister:innen oder Amtsleitungen) definiert. CA können durch ihre Entscheidungen zur bewegungsfreundlichen Gestaltung des Lebensumfelds (z. B. dem Bau eines Radwegs) das individuelle Bewegungsverhalten der Bevölkerung beeinflussen. Damit werden die Entscheidungstragenden aus Kommunalpolitik und -verwaltung zur Zielgruppe von Interventionen [3] und sollten sowohl mit kollektiven (z. B. Agenda-Setting, Organisationsentwicklung) als auch individuellen Interventionsmethoden und -strategien (z. B. persuasive Kommunikation zur Veränderung zentraler Überzeugungen) von Themen und Projekten der Gesundheits- und Bewegungsförderung überzeugt werden [21].

Es braucht jedoch in jeder Kommune Personen, die sich um die systematische Planung und Umsetzung verhältnisorientierter Interventionen kümmern und die intersektorale bzw. interdisziplinäre Zusammenarbeit verschiedener kommunaler Akteur:innen fördern [25]. Als regionale Koordinator:innen für solche Prozesse werden Akteur:innen aus dem Gesundheitsbereich bzw. öffentlichen Gesundheitsdienst (z. B. Mitarbeitende in Gesundheitsämtern oder kommunalen Gesundheitskonferenzen) vorgeschlagen [7, 17, 37]. Daneben scheinen Akteur:innen auf Landesebene sowie Kümmer:innen in der Gemeinde bzw. dem Quartier eine wichtige Rolle zu spielen [38]. Diese Koordinator:innen bzw. Akteur:innen werden im weiteren Verlauf des Beitrags als Multiplikator:innen bezeichnet. Multiplikator:innen sind alle professionellen Akteur:innen, die Prozesse und/oder Projekte der kommunalen Gesundheits- und Bewegungsförderung initiieren, planen, weitertragen oder implementieren [25]. Sie können in drei verschiedene Ebenen unterteilt werden:

1. Landesebene: Multiplikator:innen, die Prozesse/Projekte innerhalb eines Bundeslandes auf verschiedene Landkreise oder kreisfreie Städte übertragen können (z. B. Landesämter und Landesvereinigungen für Gesundheit),
2. Ebene der Landkreise und kreisfreien Städte: Multiplikator:innen, die Prozesse/Projekte auf verschiedene Gemeinden innerhalb des Landkreises oder Stadtteile innerhalb einer Stadt übertragen können (z. B. Kommunale Gesundheitskonferenzen, Mitarbeitende in Gesundheitsämtern),
3. Gemeinde- und Quartiersebene: Multiplikator:innen, die vor Ort Prozesse/Projekte umsetzen können und direkt mit der Bevölkerung interagieren (z. B. Quartiermanagements; [25]).

Das Forschungsprojekt „Entscheidungs- und Umsetzungsprozesse verhältnisorientierter Bewegungsförderung in der Kommune für mehr Chancengerechtigkeit systematisch planen und implementieren“ (EUBeKo) nimmt sowohl die kommunalen CA als auch Multiplikator:innen in den Blick und orientiert sich dabei an dem systematischen und theoriegeleiteten Vorgehen des „intervention mapping“ (u. a. Analyse des Problems, Ableitung einer Erklär- und Interventionstheorie auf Umweltebene). Dadurch werden einerseits wichtige Qualitätskriterien bei der Konzipierung, Implementierung und Evaluation verhältnisorientierter Bewegungsförderung in kommunalen Räumen berücksichtigt [23]. Andererseits können so kommunale Entscheidungsprozesse und Einflussfaktoren auf das Entscheidungsverhalten von CA als eine zentrale Voraussetzung für Verhältnisprävention in ihrem Wirkungsmechanismus verstanden und für Multiplikator:innen der Bewegungsförderung in der Kommune aufbereitet werden. Damit wird dazu beigetragen, die Replizierbarkeit, den logischen Aufbau von Interventionsansätzen sowie die Effektivität von Maßnahmen der kommunalen Gesundheits- und Bewegungsförderung zu erhöhen [10, 26].

Mit diesem Beitrag soll das Projekt EUBeKo mit seinen zwei wissenschaftlichen Forschungsfragen sowie die Planung und Umsetzung verhältnisorientierter Bewegungsförderung in zwei Modellkommunen beschrieben werden. Dabei werden die Stärken und Herausforderungen des Projekts betrachtet und eine innovative Perspektive auf die Entwicklung verhältnisorientierter Bewegungsförderung gegeben.

## Kurzbeschreibung und Ziele des Projekts

Das Projekt EUBeKo wurde vom Bundesministerium für Gesundheit vom 01.06.2019 bis zum 31.12.2022 gefördert und war als Kooperationsprojekt zwischen der Pädagogischen Hochschule Heidelberg und der Julius-Maximilians-Universität Würzburg angelegt. Ziel des Projekts war es, die Möglichkeiten von Multiplikator:innen in ländlichen und städtischen Gemeinden zum Aufbau bewegungsfördernder Strukturen zu verbessern, um langfristig eine Optimierung der Lebensverhältnisse der Bevölkerung zu erzielen. Hierzu wurden in zwei Modellkommunen in Baden-Württemberg (Mannheim-Schönau; Quartier Nordwest; städtischer Raum) und Bayern (Wülfershausen a. d. Saale; ländlicher Raum) unter Anwendung eines breiten Spektrums an Forschungsmethoden zwei zentrale Forschungsfragen bearbeitet sowie Interventionen verhältnisorientierter Bewegungsförderung geplant und umgesetzt. Das Projekt lässt sich demnach in die folgenden drei Bausteine aufteilen:

1. Baustein 1 (Forschungsfrage 1): Welches Rollenverständnis haben Multiplikator:innen der kommunalen Bewegungsförderung auf den unterschiedlichen Ebenen (z. B. Mitarbeitende in Gesundheitsämtern) im Rahmen des Planungsprozesses der verhältnisorientierten Bewegungsförderung, was sind ihre zentralen Kompetenzen und was benötigen sie zur Umsetzung eines theoriegeleiteten, systematischen Interventionsplanungs- und Implementierungsprozesses?
2. Baustein 2 (Forschungsfrage 2): Welche Einflussfaktoren (Determinanten) beeinflussen das Entscheidungsverhalten von Entscheidungstragenden aus Kommunalpolitik und -verwaltung (CA) und somit den kommunalen Entscheidungsprozess, um verhältnisorientierte Bewegungsförderung in Kommunen umsetzen zu können?
3. Baustein 3: Planung und Umsetzung verhältnisorientierter Bewegungsförderung in zwei Modellkommunen unter Begleitung der beiden Hochschulteams, um bauliche und strukturelle Veränderungen einzuleiten, die das Bewegungsverhalten der Bewohner:innen vor Ort fördern.

## Projektverlauf

**Abb. 1:**
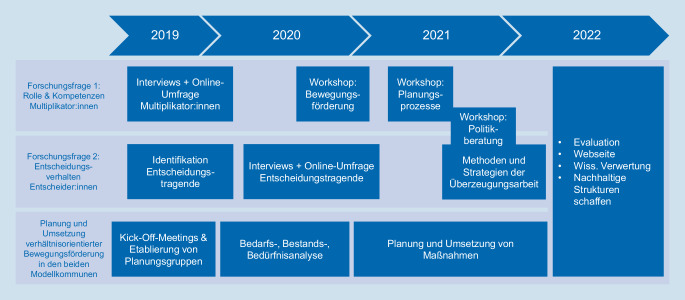
Verlauf des Projekts EUBeKo (Entscheidungs- und Umsetzungsprozesse verhältnisorientierter Bewegungsförderung in der Kommune für mehr Chancengerechtigkeit systematisch planen und implementieren). (Eigene Darstellung)

Nachfolgend wird das Projekt EUBeKo entlang seiner drei Bausteine, bestehend aus den beiden Forschungsfragen sowie der Planung und Umsetzung verhältnisorientierter Bewegungsförderung in den beiden Modellkommunen, näher beschrieben (Abb. 1).

### Baustein 1: Forschungsfrage 1: Rolle und Kompetenzen von Multiplikator:innen

In 2019 wurden für die erste Forschungsfrage ein Interviewleitfaden und eine Online-Befragung entwickelt, um das Rollenverständnis und den Ist-Zustand der Kompetenzen von Multiplikator:innen der kommunalen Bewegungsförderung aus den beiden Modellkommunen zu erfassen. Die Interviewdurchführung fand inklusive Pretests zwischen Dezember 2019 und Februar 2020 statt. Es wurden insgesamt 18 Interviews geführt, welche anschließend mittels der Software MAXQDA 2020 (VERBI GmbH, Berlin, Deutschland) kodiert und in Anlehnung an die qualitative Inhaltsanalyse nach Kuckartz [22] ausgewertet wurden. An der Online-Befragung nahmen 57 Personen teil. Diese wurde deskriptiv mittels Microsoft Excel 2016 (Microsoft Corporation, Redmond, WA, USA) und IBM SPSS Statistics 26 (IBM Corporation, Armonk, NY, USA) ausgewertet. Bei der Befragung hatten die Multiplikator:innen außerdem die Aufgabe, relevante Entscheider:innen für die Umsetzung kommunaler Gesundheits- und Bewegungsförderung (bspw. anhand einer Stakeholder-Analyse) zu identifizieren [25].

Daraufhin wurden drei Workshops zur Kompetenzerweiterung der Multiplikator:innen konzipiert, die sich inhaltlich am identifizierten Bedarf durch die Interviews und Online-Befragung orientierten. So wurden im Oktober 2020 ein Workshop zu Bewegung und Bewegungsförderung, im Juli 2021 zu Planungsprozessen (im Videoformat) und im September 2021 zu Lobbyarbeit und Politikberatung für die Mitglieder der Planungsgruppen aus beiden Modellkommunen angeboten.

Die Kompetenzerweiterung der Multiplikator:innen wurde anschließend mittels der qualitativen Methode Fokusgruppe im März 2022 untersucht. Hierbei wurde eruiert, welche Erkenntnisse die Multiplikator:innen durch die Teilnahme an den Workshops gewonnen hatten und was sich durch die Teilnahme an den Workshops in ihrer Arbeit verändert hatte.

### Baustein 2: Forschungsfrage 2: Entscheidungsverhalten von Entscheider:innen

Im Rahmen der zweiten Forschungsfrage wurden nach der Identifikation kommunaler Entscheidungstragender durch die Multiplikator:innen (s. Forschungsfrage 1) im Frühjahr 2020 ein Interviewleitfaden und eine Online-Befragung zu Entscheidungsprozessen und dem Entscheidungsverhalten von Entscheidungstragenden in der Kommune entwickelt.

Daraufhin wurden leitfadengestützte Interviews mit 22 Entscheider:innen aus Kommunalpolitik und -verwaltung im Zeitraum von Juli bis Dezember 2020 geführt, um Einblicke in kommunale Entscheidungsprozesse zu erhalten und Einflussfaktoren auf das Entscheidungsverhalten zu identifizieren. Hier ging es u. a. darum, bestimmte Einstellungen, Überzeugungen und Selbstwirksamkeitserwartungen sowie förderliche und hinderliche Faktoren zu identifizieren, um Veränderungen in der kommunalen Bewegungsumwelt umsetzen zu können. Danach wurde zwischen Januar und März 2021 eine bundesweite Online-Befragung zu den gleichen Themen durchgeführt. An der Online-Befragung nahmen insgesamt 415 Personen teil. Die Interviews wurden anschließend mittels der Software MAXQDA 2020 und in Anlehnung an die qualitative Inhaltsanalyse nach Kuckartz [22] ausgewertet. Bei der Online-Befragung kam eine deskriptive Auswertung mittels Excel und SPSS zum Tragen.

Anschließend wurden aus den Interviews und der Online-Befragung Interventionsmethoden und -strategien der Überzeugungsarbeit (in Anlehnung an „intervention mapping“ Schritt 3, [3]) abgeleitet und ein Leitfaden mit praktischen Hinweisen zur Lobbyarbeit erstellt (verfügbar unter www.gesunde-bewegte-kommune.de). Darüber hinaus wurde anhand eines Kurzfragebogens ein:e Entscheidungstragende:r zur geleisteten Überzeugungsarbeit eines Planungsgruppenmitglieds befragt. Zudem wurden in einem Workshop vergangene, gegenwärtige und zukünftige Entscheidungsprozesse aufgearbeitet und gemeinsam mit den Multiplikator:innen die praktische Anwendung von Methoden und Strategien der Überzeugungsarbeit diskutiert.

### Baustein 3: Planung und Umsetzung verhältnisorientierter Bewegungsförderung in den beiden Modellkommunen

**Abb. 2:**
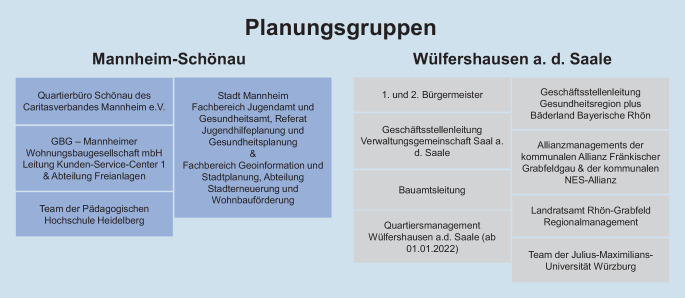
Zusammensetzung der Planungsgruppen in den beiden Modellkommunen. (Eigene Darstellung)

Im Herbst 2019 wurden drei Kick-off-Veranstaltungen mit dem Landesgesundheitsamt Baden-Württemberg und dem Bayerischen Landesamt für Lebensmittelsicherheit und Gesundheit sowie jeweils mit Akteur:innen der beiden Modellkommunen Mannheim-Schönau und Wülfershausen a. d. Saale durchgeführt. Die Kooperation mit den beiden Landesämtern stellte eine wertvolle Verbindung für die Multiplikation und Verbreitung des Projekts in den Bundesländern dar. In den Kick-off-Meetings mit den Modellkommunen wurden intersektoral und interdisziplinär zusammengesetzte Planungsgruppen etabliert (Abb. 2), welche sich ca. einmal im Quartal trafen, um die Interventionen vor Ort partizipativ zu planen und umzusetzen. Darüber hinaus fanden gemeinsame Planungsgruppentreffen mit beiden Modellkommunen statt, um gemeinsam den Projektfortschritt zu reflektieren und einen Austausch zwischen den beiden Kommunen zu gewährleisten.

Anschließend wurden kommunale Daten der beiden Modellkommunen (bspw. durch Asset-Analysen) analysiert sowie nationale Daten zum Bewegungsverhalten, zu sozialökologischen Determinanten und zu kommunalen Interventionsprogrammen über einschlägige Datenbanken (z. B. PubMed) identifiziert und aufbereitet. Darüber hinaus wurden Stakeholder-Analysen durchgeführt, um alle relevanten kommunalen Akteur:innen für das Projekt zu identifizieren und die Planungsgruppen gegebenenfalls um diese zu erweitern. Zudem wurden erste Bürger:innenbeteiligungsverfahren (z. B. Stadtteilspaziergang in Mannheim-Schönau; World Café in Wülfershausen a. d. Saale) zur Identifikation der Problemstellen und zum Stellenwert von Bewegung in den Modellkommunen durchgeführt.

Daneben wurden folgende Instrumente zur Erfassung der kommunalen Bewegungsverhältnisse und des Bewegungsverhaltens der Bewohner:innen identifiziert, verglichen, ausgewählt und für die Anwendung in den beiden Modellkommunen angepasst:

- Fragebögen für Kinder, Jugendliche und Erwachsene (u. a. Gesundheitsbefragung „Gesundheit in Deutschland aktuell“ des Robert Koch-Instituts mit dem europäischen Fragebogen „European Health Interview Survey“ (GEDA 2014/2015-EHIS [31], Assessing Levels of Physical Activity and Fitness at Population Level [ALPHA], [36])),
- Audits bzw. Begehungsprotokolle: Microscale Audit of Pedestrian Streetscapes (MAPS) Global [9] und Rural Active Living Assessment (RALA; [41]),
- Bürger:innenbeteiligungsverfahren: Photovoice, Nadelmethode, Gesprächsnachmittage mit Kindern, Jugendlichen und Senior:innen.

Für Schönau-Nordwest, einem sozialstrukturell schwachen Quartier des Stadtteils Mannheim-Schönau, wurden die Fragebögen außerdem auf polnisch, bulgarisch, türkisch und rumänisch übersetzt, um Personen mit Migrationshintergrund zu erreichen.

Insgesamt nahmen in Schönau-Nordwest 5,5 % der 2769 Einwohner:innen an der schriftlichen Befragung teil; davon 34 Kinder (Beantwortung der Fragen durch einen Elternteil), 22 Jugendliche und 96 Erwachsene. In Wülfershausen a. d. Saale wurden zusammen mit dem Gemeindeteil Eichenhausen 12 % der 1449 Einwohner:innen erreicht; davon 18 Kinder (Beantwortung der Fragen durch einen Elternteil), 16 Jugendliche und 139 Erwachsene. Zusätzlich wurden die Modellkommunen mit Hilfe der Instrumente MAPS Global (Mannheim-Schönau) und RALA (Wülfershausen a. d. Saale) zusammen mit den Planungsgruppenmitgliedern auditiert, um die baulich-technische und natürliche Bewegungsumwelt der Bewohner:innen zu erfassen. Darüber hinaus fanden weitere Bürger:innenbeteiligungsverfahren statt, z. B. Gesprächsnachmittage mit Senior:innen sowie mit Kindern und Jugendlichen, u. a. mit Hilfe der Nadelmethode.

Die Analyseergebnisse wurden nach Handlungsfeldern gegliedert in Tabellenform zusammengefasst und aufbereitet. Die Handlungsfelder wurden aus wissenschaftlichen Erkenntnissen zu Merkmalen gesunder und bewegungsfreundlicher Kommunen [6, 18] abgeleitet. Die aufbereiteten Ergebnisse wurden dem Bezirksbeirat Mannheim-Schönau und dem Gemeinderat Wülfershausen a. d. Saale vorgestellt. Der Gemeinderat Wülfershausen a. d. Saale nahm eine Priorisierung der Handlungsfelder vor und entschied, dass die Handlungsfelder „Begegnungsorte“, „Sport- und Freizeitanlagen“ und „Naturräume“ prioritär bearbeitet werden sollten. Anschließend identifizierten die beiden Planungsgruppen auf Grundlage der Analyseergebnisse verhältnisorientierte Maßnahmen und formulierten konkrete Handlungsziele, um Maßnahmen zur kommunalen Bewegungsförderung umzusetzen: den „Schönauer Rundweg“ in Mannheim und die „Bewegte Dorfrunde“ in Wülfershausen a. d. Saale.

In Mannheim-Schönau wurden die Bedürfnisse der Bevölkerung sowie Vorschläge aus wissenschaftlicher Literatur bei der Gestaltung eines quartierverbindenden Rundwegs aufgenommen. So wurden beispielsweise Bodenbeläge und Sitzmöglichkeiten sowie angrenzende Bewegungsflächen mit Bewegungselementen entlang des „Schönauer Rundwegs“ geplant und ein erstes Teilstück unter Beteiligung der Bevölkerung und Kommunalpolitik im Sommer 2021 eröffnet. Darüber hinaus wurden Flyer mit Bewegungsangeboten im Stadtteil für verschiedene Zielgruppen erstellt. In Wülfershausen a. d. Saale wurden wesentliche Schritte für die Projektmaßnahme „Bewegte Dorfrunde“ durchgeführt: Standorte für einzelne Stationen und Sitzgelegenheiten wurden identifiziert, eine Arbeitsgruppe bestehend aus Vertreter:innen unterschiedlicher Zielgruppen aus der Gemeinde gegründet und Überlegungen getroffen, wie die Bevölkerung zur Nutzung der Dorfrunde aktiviert werden könnte. Bei der „Bewegten Dorfrunde“ handelt es sich um einen beschilderten Gemeinderundweg, der mit Hilfe niedrigschwelliger, motivierender und möglichst barrierefreier Stationen alle Generationen zu Bewegung, Spiel und Spaß animieren und durch neu geschaffene Sitzmöglichkeiten auch interaktions- und begegnungsförderlich wirkt. Die „Bewegte Dorfrunde“ in Wülfershausen a. d. Saale wurde im Sommer 2022 eröffnet und im zugehörigen Ortsteil Eichenhausen ist eine weitere in Planung. Darüber hinaus wurde ein Bikepark initiiert und eröffnet und eine Neugestaltung des Kreuzungsbereichs an der Bundesstraße B279 in die Wege geleitet. Alle Umsetzungsschritte einschließlich Abweichungen und Umsetzungsbarrieren wurden prozessbegleitend dokumentiert.

Veränderungen, Ergebnisse und die Projektarbeit in den Modellkommunen wurden im September 2022 in einem Treffen mit beiden Planungsgruppen festgehalten. Darüber hinaus wurden die Wünsche und Bedürfnisse von Kindern und Jugendlichen in Mannheim-Schönau zum aktuellen Planungsstand des Rundwegs mittels Photovoice erfasst. Die Projektmaßnahme „Bewegte Dorfrunde“ in Wülfershausen a. d. Saale wurde zudem bezüglich der Zielgruppenerreichung mittels Fragebogen evaluiert.

Im Sinne der Nachhaltigkeit wurden Strukturen in den beiden Modellkommunen geschaffen. So bewirkt bspw. die physische Bebauung („Bewegte Dorfrunde“, „Schönauer Rundweg“) eine nachhaltige räumliche Veränderung in den Kommunen. Zu Projektende wurden außerdem die Finanzierung, Pflege und Wartung der „Bewegten Dorfrunde“ und des „Schönauer Rundwegs“ gesichert. Zudem bleibt die intersektorale und interdisziplinäre Zusammenarbeit in Form der Planungsgruppen oder durch die Integration in andere Gremien bestehen, um weiterhin das Thema Bewegungsförderung zu bearbeiten, auch wenn die beiden Hochschulteams die Modellkommunen nach Projektende nicht mehr begleiten können. Darüber hinaus soll im Landkreis Rhön-Grabfeld ein Netzwerk mit Gesundheitsbotschafter:innen [33] in jeder Gemeinde entstehen, welche als Bindeglied zwischen Gesundheitsbelangen der Gemeinden und der Gesundheitsregion^plus^ fungieren sollen.

Detaillierte Informationen zu den beiden Praxisbeispielen aus Mannheim-Schönau und Wülfershausen a. d. Saale finden sich unter www.gesunde-bewegte-kommune.de.

Eine Dissemination der Projektergebnisse findet sich in unterschiedlichen projektbezogenen Publikationen, welche entweder bereits veröffentlicht [25] oder im Entstehen sind, sowie auf der Projekt-Webseite www.gesunde-bewegte-kommune.de. Darüber hinaus fand von Beginn des Projekts an eine kontinuierliche Öffentlichkeitsarbeit zu den Projektaktivtäten in den beiden Modellkommunen (bspw. Gemeindeblatt, regionale Zeitungen, Facebook, Instagram) statt.

## Stärken und Herausforderungen des Projekts

Zu den Stärken des Projekts zählt aus wissenschaftlicher Sicht die systematische und theoriegeleitete Aufbereitung und Durchführung eines Prozesses verhältnisorientierter Bewegungsförderung in der Kommune in Anlehnung an das Planungsmodell „intervention mapping“ [3] mit besonderem Fokus auf Planungs- und Entscheidungsprozesse sowie auf die Zielgruppen der kommunalen Multiplikator:innen und Entscheidungstragenden.

Auf Grundlage der Ergebnisse der Befragungen mit den Multiplikator:innen kommunaler Bewegungsförderung wurden zum einen Rollen, Verantwortlichkeiten und Zuständigkeiten geklärt sowie zum anderen die benötigten Kenntnisse, Fähigkeiten und Kompetenzen in Bezug auf verhältnisorientierte Bewegungsförderung erhoben und die Multiplikator:innen mit Hilfe von Workshops zur systematischen Planung und Durchführung von verhältnisorientierter Bewegungsförderung sowie zur Politikberatung und Lobbyarbeit befähigt. Die Materialien und Ergebnisse der Workshops fließen wiederum in die Projekt-Webseite ein.

Die Ergebnisse der Erhebungen der Einflussfaktoren auf kommunale Entscheidungen geben einen ersten Einblick in diese Thematik aus gesundheitswissenschaftlicher Perspektive und bieten einen Ausgangspunkt für Anschlussprojekte zur weitergehenden Untersuchung der effektivsten Methoden und Strategien zur Überzeugung von kommunalen Entscheider:innen. Die Forschungsarbeit in diesem Bereich ist eine der ersten, die CA aus Kommunalpolitik und -verwaltung fokussiert und sie hebt die Komplexität kommunaler Entscheidungsprozesse und des Entscheidungsverhaltens von CA hervor. Es lohnt sich, mehr über die politischen Prozesse in Kommunen zu lernen und darüber, inwiefern relevante Entscheidungen für Gesundheits- und Bewegungsförderung beeinflusst werden können. Die Erkenntnisse dienen zum einen der Förderung der politischen Unterstützung und Fürsprache für zukünftige Projekte und Prozesse in den Bereichen kommunaler Gesundheits- und Bewegungsförderung, zum anderen stellen die Ergebnisse auch Informationen für andere Settings, wie Schulen oder Betriebe, zur Verfügung. Zudem dienen die Ergebnisse als Basis, um Entscheidungsverhalten in ein logisches Modell der Veränderung zu bringen und sind damit ein Ausgangspunkt, um das sozialökologische Bedingungsgefüge des Entscheidungsverhaltens von CA besser zu verstehen [3].

Auf Ebene der Planung und Umsetzung kommunaler Bewegungsförderung in den Modellkommunen ist eine Stärke des Projekts, dass sowohl die intersektorale, interdisziplinäre Zusammenarbeit durch die Etablierung von Planungsgruppen, als auch die Partizipation ihrer Mitglieder gefördert und somit Planungs- und Implementierungsprozesse besser verstanden wurden. Innerhalb der Planungsgruppen tauschten sich die Multiplikator:innen aus und bestimmen aktiv über den weiteren Projektverlauf mit. Darüber hinaus wurden in beiden Modellkommunen verschiedene Erhebungsmethoden und Bürger:innenbeteiligungsverfahren sowie die systematische Planung und Umsetzung verhältnisorientierter Bewegungsförderung erprobt. Die Erkenntnisse können anderen Kommunen als Vorlage dienen.

Die größte Herausforderung des Projekts EUBeKo ist der Theorie-Praxis-Transfer, also die Übertragung eines systematischen Planungsprozesses in die tägliche Arbeit der Akteur:innen in den Modellkommunen, um komplexe Gesundheitsprobleme zu adressieren bzw. um verhältnisorientierte Bewegungsförderung zu initiieren. Dabei ist es gerade hier wichtig, die Stärken von Wissenschaft (Hochschulteams) und Praxis (kommunalen Akteur:innen) zusammenzubringen [15]. Bis heute wurden klassische Planungsmodelle, wie das „intervention mapping“ häufig von Wissenschaftler:innen eingesetzt und weniger kommunale Akteur:innen oder die Bevölkerung involviert, daher können Adaptionen der Originalversion des Planungsmodells nötig sein, um den Prozess für alle Beteiligten handhabbar und realistisch zu halten (AIM-Modell, „adapted version of intervention mapping“, [5]). In EUBeKo wurde in diesem Zusammenhang ebenfalls deutlich, dass die Umsetzung abstrakter und theoriegeleiteter Planungsmodelle nicht originalgetreu möglich ist und eine gewisse Vorarbeit benötigt wird (z. B. in der Erhebung des gesundheitlichen Problems und der Aufstellung einer Theorie des Problems oder Intervention; [3]), während in der Praxis Prozesse intuitiver und schneller ablaufen. Zum anderen wird der Mehrwert systematischer Planung in der Praxis nicht priorisiert und als Mehraufwand gedeutet. Zudem gibt es in der Praxis meist vorab konkrete Vorstellungen über Projektziele, den weiteren Projektverlauf und den gespürten Druck, sichtbare Ergebnisse für die Bevölkerung zeitnah zu liefern. Währenddessen gehen Planungsmodelle wie das „intervention mapping“ ergebnisoffener und mehrstufiger vor, und der Fokus liegt stärker auf dem objektiven Bedarf, den Bedürfnissen der Bevölkerung und daraus resultierend einer theoriegeleiteten Planung von Interventionen [3]. Es ist jedoch wichtig, anzuerkennen, dass kommunale Akteur:innen aus der Praxis bereits unter einem hohen Druck stehen, um ihre alltäglichen Aufgaben zu erfüllen und diese Aufgaben oftmals keinen Schwerpunkt auf Gesundheits- und Bewegungsförderung legen. So bedeutet es für die Akteur:innen einen Mehraufwand, genau diesen Themen Aufmerksamkeit zu schenken. Nichtsdestotrotz bedeutet das Zusammenspiel aus Wissenschaft und Praxis einen Mehrwert in der Schaffung gesünderer Kommunen und anderer Lebenswelten [5].

Eine weitere Herausforderung ist die Auswirkung der COVID-19-Pandemie („coronavirus disease 2019“) auf die Projektumsetzung. Zu Beginn der COVID-19-Pandemie wurden Befragungen mit der Bevölkerung in den beiden Modellkommunen sowie mit den Entscheidungstragenden geplant und teilweise bereits umgesetzt. Pandemiebedingt hatten sich jedoch einige Erhebungen verschoben und damit auch die Maßnahmenumsetzung. Zudem konnten vermutlich weniger Einwohner:innen bei den Befragungen und Bürger:innenbeteilgungsverfahren erreicht werden, da die Pandemie eine allgemeine hohe Unsicherheit und Angst bei öffentlichen Treffen aufgrund des Infektionsrisikos und der noch nicht vorhandenen Impfmöglichkeiten mit sich brachte. Da das Projekt EUBeKo u. a. mit Kommunalverwaltungen kooperierte, kam es zeitweise aufgrund neuer und zusätzlicher Aufgaben der Planungsgruppenmitglieder zu zeitlichen Engpässen, um an den Planungsgruppentreffen teilzunehmen. Trotz der vielschichtigen Herausforderungen, die die COVID-19-Pandemie mit sich brachte, sind neue Handlungsmöglichkeiten entstanden. So konnten beispielweise Treffen zwischen den Hochschulteams und mit den Planungsgruppen in ein digitales Format überführt werden, was zur Zeit- und Kostenersparnis bei Anreisen als auch zur flexiblen Terminfindung beigetragen hat. Planungsgruppentreffen wurden daraufhin gleichermaßen digital und in Präsenz terminiert, um gemeinsam an der Maßnahmenumsetzung weiterzuarbeiten und um eine nachhaltige Verankerung bewegungsförderlicher Strukturen in den Modellkommunen zu erreichen.

## Nutzen für die Praxis und Ausblick

Das Projekt EUBeKo bietet vielfältige Ergebnisse und Produkte, die über das Projektende hinaus genutzt werden können, um verhältnisorientierte Bewegungsförderung in Kommunen zu fördern und multiplizieren. Zu erwähnen ist hier v. a. die Projekt-Webseite www.gesunde-bewegte-kommune.de zur kommunalen Gesundheits- und Bewegungsförderung für Praktiker:innen, welche in Zusammenarbeit mit den Planungsgruppenmitgliedern der beiden Modellkommunen erstellt wurde. Dort finden sich u. a. Informationen und praktische Tools zur systematischen Planung und Umsetzung kommunaler Gesundheits- und Bewegungsförderung, zu kommunalen Entscheidungsprozessen sowie zu den Praxisbeispielen aus Mannheim-Schönau und Wülfershausen a. d. Saale.

Darüber hinaus wurde ein Leitfaden zur Lobbyarbeit in der kommunalen Bewegungsförderung erstellt sowie Tabellen zu Interventionsmethoden und -strategien auf Beispiele der kommunalen Bewegungsförderung, angelehnt an das „intervention mapping“ [3], angepasst (beides ebenfalls abrufbar über die Webseite). Auch die Materialien und Planungen der Workshops zu den drei Themenfeldern „Kommunale Bewegungsförderung“, „Systematische Planung“ und „Lobbyarbeit und Politikberatung“ liegen vor und können jederzeit wiederverwendet werden.

Die Projektergebnisse fließen weiterhin in die Studiengangaktivitäten an der Pädagogischen Hochschule Heidelberg sowie der Julius-Maximilians-Universität Würzburg ein. Die Erfahrungen aus dem Projekt werden für die Qualifizierung zukünftiger Absolvent:innen genutzt und das Knowhow für ein evidenzgestütztes Arbeiten in der späteren Praxis und Forschung vermittelt.

Eine bundesweite digitale Abschlussveranstaltung für interessierte Multiplikator:innen der Gesundheits- und Bewegungsförderung im November 2022 rundete das Projekt ab.

## Fazit für die Praxis

- Das Projekt EUBeKo (Entscheidungs- und Umsetzungsprozesse verhältnisorientierter Bewegungsförderung in der Kommune für mehr Chancengerechtigkeit systematisch planen und implementieren) bietet mit seinem besonderen Fokus auf Multiplikator:innen der Gesundheits- und Bewegungsförderung sowie Entscheidungstragende erste Hinweise für die Planung und Umsetzung verhältnisorientierter Bewegungsförderung im Setting Kommune und kann Praktiker:innen als Orientierungspunkt dienen.
- Es müssen Strukturen und Qualifizierungsmöglichkeiten für verhältnisorientierte Bewegungsförderung in der Kommune geschaffen werden.
- Die Projekt-Webseite www.gesunde-bewegte-kommune.de bietet Praktiker:innen weiterführende Informationen und Tools für die eigene Arbeit.
